# *PLoS Computational Biology* Conference Postcards from ISMB 2010

**DOI:** 10.1371/journal.pcbi.1002000

**Published:** 2010-11-11

**Authors:** Guilhem Chalancon, Mickey Kosloff, Hatice Ulku Osmanbeyoglu, Saras Saraswathi

**Affiliations:** 1 MRC Laboratory of Molecular Biology, Cambridge, United Kingdom; 2 Ecole Normale Supérieure de Cachan, Cachan, France; 3 Duke University Medical Center, Durham, North Carolina, United States of America; 4 Department of Biomedical Informatics, University of Pittsburgh, Pittsburgh, Pennsylvania, United States of America; 5 Bioinformatics and Computational Biology Program, Iowa State University, Ames, Iowa, United States of America; University of California San Diego, United States of America

The annual international conference on Intelligent Systems for Molecular Biology (ISMB) is the largest meeting of the International Society for Computational Biology (ISCB). In 2010 it was held in Boston, United States, July 11–13. What follows are four conference postcards that reflect different activities considered exciting and important by younger attendees. Postcards, as the name suggests, are brief reports on the talks and other events that interested attendees. You can read more about the idea of conference postcards at http://www.ploscompbiol.org/doi/pcbi.1000746, and if you are a graduate student or postdoctoral fellow, please consider contributing postcards at any future meetings of interest to the *PLoS Computational Biology* readership. We want to hear your view of the science being presented.

## Robert F. Murphy on “Determining the Distribution of Probes between Different Subcellular Locations through Automated Unmixing of Subcellular Patterns” in the ISMB Highlights Session

### Reported by Guilhem Chalancon, MRC Laboratory of Molecular Biology

Many outstanding talks were given at the ISMB 2010 conference, and the work presented by Dr. Robert F. Murphy during the second day of the conference was certainly one of them. Murphy, who is a professor at Carnegie Mellon University, presented a tool called PatternUnmixer [1] that uses high-throughput automated microscopy data to quantify the distribution of fluorescently labeled proteins across different cellular compartments [2]. There is a strong need for automated and accurate acquisition of protein localization data, which requires advanced computational methods. Such tools are now even more accurate than visual analysis to describe large numbers of subcellular structures. However, proteins often localize simultaneously in several subcellular locations. Consequently, fluorescently labeled proteins often form “mixed patterns” of fluorescence, in which the signal of fluorescence is the result of two or more distinct patterns.

The method presented by Murphy aims to characterize such mixed patterns of fluorescence. It involves several steps, starting with the extraction of all the objects detected in a set of images for a single probe, which constitutes one training set. The features of the objects—such as their size, shape, and distance from the nucleus, for instance—are then collected. These features are used to classify every individual object into categories called “object types” that are defined for a cell type in a given set of conditions and for one probe. Once the object types are defined for all the probes (and their training sets), the tested set of images—which contains mixed patterns—is then investigated. The proportion of signals resulting from each pattern is estimated based on the distributions acquired from the training sets. As such, it means that one can “unmix” the patterns of fluorescence of a protein located in two (or potentially more) compartments.

To give a proof of the concept, Murphy and his team used fluorescent trackers for mitochondria and lysosomes in HeLa cells and generated a collection of images. They used these two probes to define “pure” patterns and a combination thereof in order to obtain mixed patterns, using determined concentrations of trackers. In other words, by training their method to identify objects marked by two different trackers (lysosomal or mitochondrial), they were able to unmix the two signals in cells with mixed labeling. The proportions of signal belonging to lysosomal tracker or mitochondrial tracker were found to be in good agreement with the expected fractions. This suggested that the method is effective in “unmixing” fluorescent patterns.

Using this approach, Tao Peng and colleagues were then able to estimate the accumulation of LC3 (a microtubule-associated protein) in autophagosomes upon bafilomycin treatments (BAF, vacuolar ATPase inhibitor) in RT112 cells. In this experiment, the fluorescence of eGFP-LC3 was monitored, and the two training sets corresponded to either untreated or treated cells at a high dose of BAF [2]. Peng et al. observed a sigmoidal shift in distribution of LC3 between low and high concentrations of BAF, indicating its gradual relocation. This suggests that the method can be applied to obtain quantitative measurements of protein translocation dynamics. Potentially, the approach could also be used in a high-throughput manner to quantify how protein localization can change over time in the same condition, in a range of conditions, or in different genetic backgrounds.

Overall, I found that this talk was a good illustration of how experimental biology can benefit from computational approaches. The ISMB conference encourages the development of advanced computational methods that resolve biological problems, which I believe was also exemplified by this talk. My personal feeling is that the work presented by Dr. Robert F. Murphy constitutes an important step in the high-throughput collection and analysis of protein localization data at the system level. I think that this method should not only be regarded as interesting for experimentalists who want to quantify and assign protein localization objectively, but also as a starting point to generate new testable hypotheses for computational biologists who aim to understand the impact of protein localization on system-level properties. For instance, the following questions could be addressed: what is the global extent of protein translocation upon varying conditions? Or in distinct genetic backgrounds? Can one identify recurrent spatial or temporal patterns of movements (spatio-temporal motifs) of proteins? Are there common functions, properties, or sequence motifs in proteins with similar translocation pattern? How variable are protein localization patterns at a single-cell level? Are there distinct trends in the gene expression regulation of proteins whose localization is highly dynamic compared to proteins clustered in restricted subcellular locations?

Many—if not all—of the current challenges in molecular and systems biology boil down to the generation of data that are not only wide in scope (at the omic level, or at the population level), but that are also sufficiently accurate and reproducible to establish robust interpretations. There is consequently an increasing need for automated approaches to collect information with high complexity as systems biology progresses in linking information from genotype to phenotype. Recent progress brought by next-generation sequencing keeps improving the accuracy and the depth of our knowledge on genotypic information. However, our ability to accurately and quantitatively collect and describe phenotypic information still suffers a lack of automation. This is precisely what makes the current developments in high-throughput microscopy and computational methods for image analysis very exciting. In this regard, the fast acquisition of protein localization is of great importance, as this is an important aspect to characterize the phenotype of a cell.

In short, the meeting of cell biology and systems biology can fuel many applications and open new paths of exploration for computational biologists. This talk was a good indication of how we can get there, provided an intense interaction between the cell biology and computational biology communities takes place.

## James Fraser on “The Effect of Temperature on Polysterism in Protein Crystals” in the 3DSIG Satellite Meeting

### Reported by Mickey Kosloff, Duke University Medical Center

Crystal structures are of fundamental importance to biological research in general and to computational research in particular. Because cellular functions are mostly carried out by proteins in the three-dimensional (3-D) world, and because 3-D structures give us a direct visualization of that world, crystal structures are often perceived as a gold standard when it comes to deciphering protein function. Yet, once in a while, we are reminded that the beautiful 3-D structural models in the Protein Data Bank (PDB; http://www.pdb.org/) do not always give us the whole story. The talk given by James Fraser (University of California Berkeley) at 3DSIG 2010, a satellite meeting of ISMB 2010 that focuses on structural bioinformatics and computational biophysics, did exactly that—it reminded us that there is more to crystal structures than meets the eye. Fraser talked about “the effect of temperature on polysterism in protein crystals”, or in other words, about multiple conformational ensembles that can hide behind the seemingly static view of protein crystal structures.

The 3-D models of proteins in the PDB show a snapshot of a protein's structure. Nonetheless, proteins in solution are flexible and populate ensembles of conformations. In many cases, these conformations can differ substantially and such proteins can be either 1) intrinsically unstructured proteins that, when monomeric in solution, do not have a fixed 3-D structure [3]; or 2) stabilized in two or more structurally dissimilar snapshots of the same protein (e.g., [4], [5]). Smaller motions, however, such as side-chain rearrangements or fluctuations of small loops, can be resolved in the electron density maps of a single crystal structure.

In a recent paper published in *Nature* [6], Fraser and colleagues from the Alber and Kern labs explored this theme in a particular example—the human proline isomerase cyclophilin A (CypA). Using X-ray crystallographic data collected at room temperature together with nuclear magnetic resonance analysis of CypA dynamics, they discovered a network of residues with inter-converting side-chain conformations. This network of residues with alternative conformations (a.k.a. polysterism) included part of the enzyme active site, suggesting that the conformational fluctuations play a role in the enzymatic mechanism of CypA. The authors validated this hypothesis by designing a mutation in a residue outside the active site that decreased local motions, resulting in a concomitant decrease in the catalytic activity of CypA.

A subsequent paper from the Alber lab described the automated method used to identify alternative side-chain conformation, which they named Ringer [7]. Ringer extracts alternative side-chain conformations from weak electron density peaks at levels traditionally regarded as noise. By applying Ringer to high-resolution electron density maps of 402 crystal structures, they discovered that a substantial minority of side-chains show evidence for polysterism.

In the original 2009 study, Fraser et al. observed polysterism in CypA when the structure was solved at ambient temperatures but not when the structure was solved at cryo-temperatures. This observation has far-reaching implications, because nowadays the vast majority of crystallographic diffraction data is collected at cryo-temperatures. The underlying assumption is that such low temperatures result in higher quality data without a significant effect on 3-D structure. To check this assumption, Fraser applied an extended version of Ringer to the electron density maps of a hand-picked dataset of 30 protein pairs—crystal structures of the same protein, solved at both cryogenic and ambient temperatures. This comparison revealed that local temperature-dependent structural differences such as those observed in CypA are also observed in other crystal structures. Furthermore, structures solved at cryo-temperatures were more compact—their volume shrunk by up to 6% and their interior cavities contracted. When Fraser looked at individual residues, he saw that ∼10% of these showed structural differences. These differences included altered minor side-chain states, switching of minor and major states, or sampling of a dissimilar side-chain orientation altogether. In short, Fraser's data casts doubt on the prevalent assumption that a structure determined at cryo-temperatures is equivalent to the structure of the same protein that is determined at room temperature.

Fraser gave a riveting talk that was very well received by the diverse audience that attended the 2-day 3DSIG satellite meeting at ISMB 2010 (SIG, by the way, stands for Special Interest Group). As the name of the meeting suggests, 3DSIG attracts a wide range of scientists whose research connects to 3-D structures (http://bcb.med.usherbrooke.ca/3dsig10/Program.html). In my opinion, this talk stood out among other 3DSIG presentations in its elegant fusion of experiments and computations, and discussions on the implication of these results for the use of crystal structures in computational research continued well into dinner. The evidence of polysterism, hidden within crystallographic electron density maps, and the effect of temperature on these alternative conformations add to a larger picture—that an assumption of “one sequence equals one 3-D structure” can lead to the loss of important structural and functional information.

For his excellent presentation James Fraser was awarded the Warren DeLano Structural Bioinformatics and Computational Biophysics Award. Fraser graduated with a PhD from UC Berkeley in the summer of 2010 and will continue his research as a QB3 Fellow at the University of California San Francisco in January 2011. I, for one, am planning to keep an eye on his research and see what interesting findings he discovers next.

## Mark McDowall—“PIPs: Human Protein-Protein Interaction Prediction” Poster L20

### Reported by Hatice Ulku Osmanbeyoglu, University of Pittsburgh

One of the outstanding posters at ISMB was the “PIPs: Human Protein-Protein Interaction Prediction” poster by Mark McDowall (University of Dundee). His work involved using computational techniques to predict new protein–protein interactions (PPIs) as well as developing a PPI Web site (http://www.compbio.dundee.ac.uk/pips/) with the Barton group's findings to let the scientific community explore the predictions that have been made with their system. More specifically, their PIPs framework uses a naïve Bayesian method to combine the predictive capabilities of numerous features to calculate the likelihood of interaction between two proteins. Their predictor uses features such as co-expression, orthology, domain co-occurrence, post-translational modification, and semantic similarity of Gene Ontology terms. They developed two modules that make predictions based on the topology of the predicted PPI network, and several of their predictions have been experimentally validated by external groups. Moreover, they made their predictions available at the PIPs Web site (http://www.compbio.dundee.ac.uk/pips/) so the scientific community can explore them. Users can search with a protein identifier (IPI, RefSeq, or UniProt) or a keyword. All predicted PPIs are returned in order of their likelihood of interaction. The Web site allows the user to analyze the evidence used to calculate the likelihood of interaction and provides links to external databases and publications to retrieve the source data.

PPIs play a key role in the cell functioning, signaling, and metabolic pathways, and in the facilitation of structural scaffolds in organisms. There are currently about 39,000 PPIs that have been experimentally confirmed, which corresponds to approximately only 10% of the PPIs in humans. Computational methods are essential to guide further experimental endeavors to bridge the gap. Determining protein interactions via experimental methods is costly, time consuming, and not scalable. Moreover, high-throughput methods such as yeast two-hybrid (Y2H) and mass spectrometry help to determine protein interactions. But these methods suffer from high false positive rates, and many protein interaction predictions are not shared among them. For example, of the reported interactions from Y2H in yeast, around 70% are estimated to be false positives. Moreover, only around 3% of the protein interactions are supported by more than one high-throughput method [8], [9]. In complex organisms, applying high-throughput methods to test every possible protein pair would be very costly. Computational methods are therefore necessary to complete the interactome. This poster is an example of a successful computational approach.

The poster was outstanding because it demonstrated so fully one of the primary themes of the conference, “using computational tools to leverage existing datasets to drive biological discovery”. The work originates from computational analysis of existing data, and then leads to experimental validation of computational predictions, and useful biological insights.

## 6th ISCB Student Council Symposium

### Reported by Saras Saraswathi, Iowa State University

I attended the 18th Annual International Conference on Intelligent Systems for Molecular Biology (ISMB 2010) at the Hynes Convention Center here in Boston. We had a great day at the Student Council Symposium (SCS-6) organized by the ISCB Student Council. We started off the session with something called “speed dating”, which was a whole lot of fun. There were about 60 students from all corners of the world who got a chance to interact spontaneously with each other.

Speed dating is an event where each person has a colored bracelet on their wrist. There were 5-minute intervals when two people wearing a different color bracelet could interact and get to know about each other's research interests and tell a little bit about their background. Then the whistle goes “shriek” and you are off to talk to another person with a different colored bracelet. When I first heard about the idea, I have to admit I was very skeptical and wondered “what could be accomplished in 5 minutes?” But I was totally surprised by the energy it brought to the event for the whole day. We were no longer strangers from different continents. We felt we knew a lot of people who were sitting with us in that conference hall who shared similar interests.

It was a fun activity, where people were talking as fast as they could about where they came from, what work they were currently doing, and so on. Everyone was in a big hurry to finish saying what they wanted in 5 minutes if they did not intend to let the other person talk or, in 2.5 minutes, if they were willing to let the other person say something. We do have to give the other party a chance to say something too, right? But some people got carried away, while others did not feel like interrupting. This experience built social skills in learning how to present your work in a concise manner, being conscious of giving the same opportunity to the other person as one would like to have, how to get a word in when the other person does not seem to be aware of your needs to share your research, and a chance to size up their personality in a few minutes. These are essential skills one should develop to be successful in life. Since 25 to 30 conversations were going on at the same time, there was this big buzz that got louder and louder, of course! Amazingly, people took to it like fish to water, since the organizers did not have to do much in the way of facilitating it other than giving initial instructions and blowing the whistle every 5 minutes. A lot of planning and preparation went into it of course, but it was so seamless that it appeared so easy, thanks to the organizers who carried it out to perfection.

In the short time available, I was able to meet students who were from Korea working in the US, from China working in the United Kingdom, a French guy working in Germany, and so on. It was a lot of fun; it was exciting, exhilarating, and exhausting, as if I really walked all those miles around the world! You had a sort of anticipation about whom you were going to meet and from which corner of the world. I was simply amazed at the variety of people I talked to in such a short time. It broke the ice, led to instant and new friendships, and swept away the inhibitions you have in approaching someone for the first time and bragging about your research, which I am sure everyone likes to do! It will hopefully lead to a few collaborations, if the attendees had a follow-up meeting during the conference or have one later, perhaps at another meeting. I felt that I would not have met any of the researchers I chatted with, were it not for this speed dating event.

Later on at the business meeting of the Student Council, Burkhard Rost, ISCB President, was excited to hear about the speed-dating event and asked if this event could be arranged on a larger scale for 1,000 people at the next ISMB conference. So, for those who are planning to attend, get your 5 minute pitch ready—or better still, a pitch for 2.5 minutes to share your experiences.

So, was the event a success? I think it was, but there is always room for improvement. But let us hear from the participants themselves. This is what some of the participants had to say about the speed dating event (in the order in which I spoke to them):

Fadi Towfic from Iowa State University, US: “A great way to meet a lot of people in a very short time…But the time was too short to find what the other person was doing in their research area…it was a very good experience though.”

Shweta Shah from Carnegie Mellon University, US: “It was a great experience and you get to meet a lot of new people whom you will not meet otherwise…I would like to do something like this in future conferences.”

Adriana Munoz from University of Ottawa, Canada: “I think it is a very good idea and it was interesting to meet people. It made it easy for me to meet others…Great.”

Jelle ten Hoeve from Netherlands Cancer Institute, The Netherlands: “I really liked it…I met three very interesting people and the funny thing was I heard all about them, but I could not get to talk about myself… It is perfect though.”

Bastian Van den Berg from Delft University of Technology, The Netherlands: “Very nice and easy way to get in touch with PhD students…I was able to explain my project to other students.”
